# Knowledge and Lifestyle-Associated Prevalence of Obesity among Newly Diagnosed Type II Diabetes Mellitus Patients Attending Diabetic Clinic at Komfo Anokye Teaching Hospital, Kumasi, Ghana: A Hospital-Based Cross-Sectional Study

**DOI:** 10.1155/2016/9759241

**Published:** 2016-01-10

**Authors:** Yaa Obirikorang, Christian Obirikorang, Enoch Odame Anto, Emmanuel Acheampong, Nyalako Dzah, Caroline Nkrumah Akosah, Emmanuella Batu Nsenbah

**Affiliations:** ^1^Department of Nursing, Faculty of Health and Allied Sciences, Garden City University College (GCUC), Kenyasi, Kumasi, Ghana; ^2^Department of Molecular Medicine, School of Medical Science, Kwame Nkrumah University of Science and Technology (KNUST), Kumasi, Ghana; ^3^Royal Ann College of Health, Department of Medical Laboratory Technology, Atwima Manhyia, Kumasi, Ghana

## Abstract

This study aimed to determine the knowledge and prevalence of obesity among Ghanaian newly diagnosed type 2 diabetics. This cross-sectional study was conducted among diagnosed type 2 diabetics. Structured questionnaire was used to obtain data. Anthropometric measurements and fasting blood sugar levels were also assessed. Participants had adequate knowledge about the general concept of obesity (72.0%) and method of weight measurement (98.6%) but were less knowledgeable of ideal body weight (4.2%). The commonly known cause, complication, and management of obesity were poor diet (76.9%), hypertension (81.8%), and diet modification (86.7%), respectively. The anthropometric measures were higher among females compared to males. Prevalence of obesity was 61.3% according to WHR classification, 40.8% according to WHtR classification, 26.1% according to WC, and 14.8% according to BMI classification. Being female was significantly associated with high prevalence of obesity irrespective of the anthropometric measure used (*p* < 0.05). Taking of snacks in meals, eating meals late at night, physical inactivity, excessive fast food intake, and alcoholic beverage intake were associated with increased prevalence of obesity (*p* < 0.05). Prevalence of obesity is high among diabetic patient and thus increasing effort towards developing and making education programs by focusing on adjusting to lifestyle modifications is required.

## 1. Introduction

Diabetes is now recognized as a major chronic public health problem throughout the world and affecting a large number of people in a wide range of ethnic and economic levels in both developed and developing countries. However, it is estimated that the developing countries will bear the brunt of this epidemic in the 21st century, with 80% of all new cases of diabetes expected to appear in the developing countries like Ghana by 2025 [1] including South Asian countries. The risk of diabetes mellitus is independently associated with increasing age, modifiable factors related to rapid urban growth and changing lifestyle (i.e., obesity, sedentary lifestyle, lack of physical activity, diet, smoking, and physical and emotional stress), and nonmodifiable factors such as family history of diabetes, age, and race/ethnicity [2, 3].

Obesity in persons with diabetes is associated with poorer control of blood glucose levels, blood pressure, and cholesterol [4, 5], placing patients at higher risk for both cardiovascular and microvascular disease [6]. Obesity is a complex disorder involving appetite regulation and energy metabolism, as the excess of body fat results from an imbalance of intake and expenditure [7]. Obesity is considered a major risk factor for type 2 diabetes [8]. It has been found that the incidence of diabetes increases by a 2-3-fold factor in obese individuals when obesity is defined as 120% of ideal weight [9]. It does interfere with not only effective treatment of hyperglycemia, but also hypertension and dyslipidemia [10], cardiovascular disease, cerebrovascular disease, hyperlipidemia, increased incidence of arthritis of the hands and knees, gallbladder disease, and sleep apnea [11]. In addition to the increased risk of morbidity and mortality, obesity leads to various psychological stresses that vary from emotional distress to social stigmatization [12]. The rising prevalence of obesity in type 2 diabetes in Ghana is though not known but may be attributed to rapid urbanization and associated changes in lifestyle, such as sedentary lifestyle, higher-calorie food intake, and stressful life. However, evidence suggests that lifestyle related interventions targeting modifiable risk factors can either prevent or delay the onset of type 2 diabetes and future risk of obesity [13]. Management of obesity largely depends on patient motivation and education. These in turn can be greatly facilitated by adequate baseline data on the knowledge of patients about obesity. Knowledge is influenced by socioeconomic and cultural factors, attitude, readiness to learn, family support, and barriers to care. However, knowledge and prevalence of obesity among Ghanaian diabetic patients have received very little or no attention. This study was therefore conducted to assess the knowledge and prevalence of obesity among diabetic patients attending the diabetic clinic at the Komfo Anokye Teaching Hospital (KATH).

## 2. Materials and Methods

### 2.1. Study Design/Settings

This hospital-based cross-sectional descriptive study was conducted at the Komfo Anokye Teaching Hospital (KATH), Ghana. Komfo Anokye Teaching Hospital (KATH) is located in Kumasi, the regional capital of the Ashanti Region in Ghana with a total projected population of 4,780,380 (Ghana statistical service, 2010). It is the second largest hospital in Ghana with trained doctors, nurses, anaesthetists, health care assistants, and specialties in surgery internal medicine, obstetrical and gynaecological, child health, oncology, family medicine, and emergency medicine. The geographical location of the thousand- (1000-) bed capacity, the road network of the country, and commercial nature of Kumasi make the hospital accessible to all the areas that share the boundaries with Ashanti Region and others that are further away. The diabetic centre of the KATH is situated beneath the medicine block (D block) just between the chest clinic and diagnostic centre and behind the emergency unit of the hospital.

### 2.2. Study Population/Selection of Participants

Using a purposive sampling technique a total of 543 newly diagnosed type II diabetes (T2DM) patients have reported at the diabetic clinic from the period of October 2014 to May 2015. Diabetic patients who have been previously diagnosed as having T2DM before the study period and patients who are unable to give informed consent were excluded from the study. Quantitative research approach was the research method used to determine knowledge and prevalence of obesity among newly diagnosed T2DM patients. Structured questionnaire was used to obtain information from all study respondents. A structured questionnaire divided into four sections with open- and close-ended questions was used for this study. Section A involved questions that elicited information on sociodemographic variables of the diabetic patient such as age, occupation, marital status, economic income, levels of education, ethnicity, family type, and religion. Section B included questions on the knowledge T2DM patients have about obesity. Section C contained items that elicited information on dietary lifestyle, physical activity, and others such as alcohol intake and smoking. In Section D, questions were designed to obtain information relating to obesity measurements such as height, weight, waist circumference, and hip circumference of studied subjects.

### 2.3. Criteria for Scoring on the Knowledge of Obesity

Participants were said to have “adequate” knowledge of obesity if they responded to at least three correct answers each about causes, complication, management of obesity, and method of weight measurement, “inadequate” knowledge if they responded to at most one correct answer each to causes, complications, management of obesity, and method of weight measurement, and “no” knowledge (do not know) if they did not know anything about causes, complication, management of obesity, and method of weight measurement.

### 2.4. Anthropometric Measurements

Participants were made to stand without their sandals, bags, or anything of significant weight on the weighing scale (Seca, Hamburg, Deutschland) and against the stadiometer (Seca, Hamburg, Deutschland). The weight was read to the nearest 0.1 kilograms and recorded. The value for the height was recorded to the nearest 0.1 centimeters and then converted to meters. The body mass index (BMI) was calculated using formula (weight/height squared) and expressed in kg/m^2^. Gulick II spring-loaded measuring tape (Gays Mills, WI) was used to measure waist circumference midway between the inferior angle of the ribs and the suprailiac crest, whereas hip circumference was measured at the outermost points of the greater trochanters [14]. WHR and WHtR were recorded to the nearest 2 decimal places. WHR and WHtR were measured during first phase of sample collection.

BMI (kg/m^2^) was categorized, using the current World Health Organization (WHO) definitions. BMI of <18.5 kg/m^2^, 18.5–24.9 kg/m^2^, 25–29.9 kg/m^2^, and 30 kg/m^2^ were used to define underweight, normal, overweight, and obese cases, respectively. Waist circumference (WC) was defined for both males and females with WC <94, 94–101.9, and ≥102 cm defined as normal, overweight, and obese, respectively for males and <80, 80–87.9, and ≥88 cm defined as normal, overweight, and obese, respectively, for females. WHR was also defined for both males and females with WHR <0.90, 0.90–0.99, and ≥1.0 defined as normal, overweight, and obese, respectively, for males and <0.80, 0.80–0.84, and ≥0.85 defined as normal, overweight, and underweight, respectively, for females. With WHtR <0.5 is considered normal and ≥0.5 is considered obese [14, 15].

### 2.5. Statistical Methods

The data entry and analysis were performed using IBM statistical package for social science (SPSS) version 20. Descriptive statistics such as frequencies, percentage, and charts were used. Chi-square or Fischer's exact test statistical methods were used as appropriate. All results were confirmed at 5% level of significance. *p* value less than 0.05 was considered statistically significant difference.

### 2.6. Ethical Consideration

Approval for this study was obtained from the Committee on Human Research, Publication and Ethics of the School of Medical Sciences (SMS), Kwame Nkrumah University of Science and Technology (KNUST), and the Research and Development (R & D) Unit at KATH and the Head of Department of the Diabetes Unit (Ref-CHRPE/RC/157/13). Participation was voluntary and written informed consent was obtained from each participant.

## 3. Results

Table 1 shows general sociodemographic characteristics of type 2 diabetic patients. A total of five hundred and forty-three (543) type 2 diabetic (T2D) patients were recruited for this study. The mean age of the general type 2 diabetic (T2D) participants in this study was 51.14 ± 14.45 years. Higher proportions (42.7%) of them were between the ages of 40 and 59 years. Among the T2D participants, there were more females (57.3%) than males (42.7%). Three hundred and four (304) of them representing 55.9% were self-employed while 399 (73.4%) were married. Out of a total of 543 participants, higher proportions (53.8%) of them had low socioeconomic income, 38.4% had completed primary education, and 88.1% were Akans (Table 1).

As shown in Table 2, out of 543 participants, 391 representing 72.0% had adequate knowledge on the general understanding of obesity. Approximately, 26.0% of them had inadequate knowledge and a very few (2.1%) had no knowledge of the meaning of obesity. For ideal body weight, most (56.6%) of them had inadequate knowledge and 39.2% of them did not know about it though a very few (4.2%) of the participants had adequate knowledge. A higher proportion (98.6%) of the participants had adequate knowledge on the methods used in measuring weight while 1.4% had inadequate knowledge. Table 2 also shows the knowledge on understanding of obesity, ideal body weight, and methods of weight measurements among type 2 diabetic patients. Higher proportion (76.9%) of the participants responded that poor diet was a common cause of obesity followed by physical inactivity (67.1%), family history of obesity (56.6%), and insufficient sleep and stress (0.7%) The common known complications by the type 2 diabetic patients were hypertension (81.8%), followed by stroke (34.3%) and cancer (1.4%). Four hundred and seventy-one (471) of the participants representing 86.7% knew that adjusting to dietary modification is the best mode of managing obesity while 68.6% and 28.7% of them knew that doing regular physical activity and health check-up, respectively, could help manage obesity (Table 2).

Table 3 shows lifestyle characteristic of type 2 diabetic patients on the nutritional lifestyles, most (74.8%) of the participants ate thrice a day, 16.8% of them took snacks in between meals, and 13.3% ate at late hours. Most (62.2%) of the participants took their meals around 6 pm. Four hundred and ten (410) representing 75.5% of the participants were not physically active. Only 133 (24.5%) do regular exercise. The common type of exercise among the participants was walking (57.1%) followed by jogging (42.9%). Most (48.6%) of them did their exercise daily per week, though a very few (2.9%) did exercise once per week. Out of 543 participants four (4) participants (0.7%) were smoker while 15 (2.8%) of them had history of alcoholic beverage intake. A higher proportion (56.6%) of the participants prefer eating butter, cheese, and cream, 53.1% prefer soft drinks, 40.6% prefer fast foods, and 30.7% prefer fiber rich foods (30.7%) while 21.7% and 11.9% prefer to eat red meat and egg yolk, respectively (Table 3).

Table 4 shows the anthropometric, clinical, and FBS levels characteristic of type 2 diabetic patients. The mean weight, BMI, WC, HC, WHR, and WHtR were higher among females compared to males. There was statistically significant difference between mean BMI levels (*p* = 0.0038). Meanwhile males were significantly (*p* < 0.0001) taller (1.69 ± 0.01 m) than the female (1.61 ± 0.01 m) participants. There was no statistically significant difference in levels of SBP and DBP between males and females (*p* > 0.05). Mean levels of FBS were significantly higher in males (13.52 ± 0.93 mmol/L) compared to females (10.50 ± 0.58 mmol/L) (*p* = 0.0044) (Table 4).

Table 5 shows the prevalence of obesity according the gender. There was a significantly higher proportion of obesity among females compared to male participants. Prevalence of obesity in male compared to females was 14.3% versus 85.7% using BMI classification, 13.5% versus 86.5% using WC, 14.9% versus 85.1% using WHR, and 37.9% versus 62.1% using WHtR. The difference in proportion was statistically significant irrespective of the method used (*p* < 0.05) (Table 5).

Tables 6 and 7 show the lifestyle characteristic features in relation to prevalence of obesity classified by BMI, WC, WHR, and WHtR. For participants who took snacks in between meals the prevalence of obesity was 33.3% using BMI, 25.0% using WC, 62.5% using WHR, and 54.2% using WHtR classification. The prevalence of obesity using BMI was 31.5% for those who ate late at night, 31.6% using WC, 73.7% using WHR, and 52.6% using WHtR. Approximately, eighteen percent (17.6%) of participants who were physically inactive were obese according to the BMI classification while 27.8%, 62.0%, and 40.8% were obese when WC, WHR, and WHtR, respectively, were used. The prevalence of obesity among participants who eat fast foods was 17.2% using BMI, 29.3% using WC, 64.3% using WHR, and 55.4% using WHtR. Twenty-five percent (25.0%) of participants with history of alcohol intake were obese using the BMI, WC, and WHR whereas 75.0% were obese using WHtR (Tables 6 and 7).

Table 8 shows the association between level of knowledge of obesity and the prevalence among type 2 diabetics. Using BMI as an indicator, 21.2% of participants who had adequate knowledge, 17.7% who had inadequate knowledge, and 54.5% who had no knowledge were obese (*p* = 0.1282) while 23.3% of participants with adequate knowledge, 36.2% with inadequate knowledge, and 81.8% of those with no knowledge of obesity were obese using WC (*p* < 0.0001). Using WHR as an indicator, 19.7% of participants with adequate knowledge, 29.1% with inadequate knowledge, and 45.5% with no knowledge of obesity were obese (*p* = 0.0101) while 22.5% of participants with adequate knowledge, 39.3% with inadequate knowledge, and 72.2% with no knowledge of obesity were obese (*p* < 0.0001).

## 4. Discussion 

Globally, over 300 million and 1.1 billion cases of adult obesity and overweight are reported annually [8]. For the first time, this study assessed the knowledge and prevalence of obesity among newly diagnosed type 2 diabetic patients at the Komfo Anokye Teaching Hospital, Kumasi, Ghana. In this study, 72.0% of the respondents had adequate knowledge about general concept of obesity and 98.6% were knowledgeable of the weight measurement technique while only 4.2% had adequate knowledge of their ideal body weight. In a study by Qidwai and Azam [16] majority of the participants were well informed on the general concept of obesity which is consistent with the finding of this study. Again, the findings that participants had adequate knowledge of weight measurement techniques are not consistent with findings by Saleh et al. [17]. Conversely, 56.6% had inadequate knowledge about ideal body weight which is consistent with Saleh et al. [17].

Moreover, when knowledge of diabetic patients was assessed on causes, complications, and management of obesity, most of the participants knew that poor dietary habit is a major cause obesity and also hypertension and stroke were the commonly known complications of obesity. Dietary modification and regular physical activity were the common management approaches of obesity known by participants.

Furthermore, participants were asked about the kind of food they considered healthy. Majority of them considered butter, cheese, and cream (56.6%), soft drinks (53.1%), fast foods (40.6%), fiber rich foods (30.7%), red meat (21.7%), and egg yolk (11.9%) as healthier food. This is in agreement with a study by Saleh et al. [17] who reported that majority of the diabetic patients preferred fast food, soft drinks, and mayonnaise as they considered them healthy food. Such eating preferences result in the development of overweight and obesity among patients and evidence suggests that reduction in the intake of fat and sugar leads to body weight control and prevents overweight and obesity [18]. Similar study conducted in Karachi, Pakistan, also showed that a large proportion of participants preferred oily and fried food [16]. The need for education in these areas is required. Badruddin et al. reported that dietary advice should be given to individuals with clear view of its purpose, so that they can understand and follow it in practice [19]. Therefore the role of appropriate dietary measures to control bodyweight is extremely important. It is encouraging to note that majority of the respondents believe that dietary modification is the first line of management to obesity. If such belief could be transformed into practice, then a reduced future risk of cardiovascular disease will be an achievable target.

This study also assessed several lifestyle characteristics of diabetic patients. The common lifestyle behaviours were taking snacks in between meals (16.8%), eating at late hours in the night (13.3%), regular physical exercise (24.5%), smoking (0.7%), and alcoholic intake (2.8%). From this study, less than 30% of the diabetic patients did regular physical exercise and the most common forms were walking (57.1%) and jogging (42.9%). This proportion of participants in this study who did regular exercise is low compared to 59% reported by Qidwai and Azam [16]. It has been shown that reduced levels of physical activity play a predominant role in the development of obesity [20]. Therefore there is need for education on the importance of exercise and also on the need to exercise for certain duration and at the optimum frequency. Several studies conducted in urbanizing rural community of Bangladesh showed that there is a significant association between higher body mass index (BMI) and incidence of diabetes mellitus [2–4]. However, in Ghana, published data showing the relationship between obesity and newly diagnosed diabetic patient is scarce. Aside the paucity of data, majority of these studies focus on using only BMI as a measure of obesity. In this study, obesity was assessed by BMI, WC, WHR, and WHtR classification. The results indicated that the prevalence of obesity among type 2 diabetic patients was 61.3% according to WHR classification, 40.8% according to WHtR classification, 26.1% according to WC, and 14.8% according to BMI classification.

The prevalence of obesity using WC, WHR, and WHtR (Table 6) was significantly higher compared to using BMI. Previous studies have also reported increased prevalence of obesity by WHR and WC [21, 22] and WHtR [23] which is inconsistent with findings of this study. Another study reported both WC and BMI as having equal diagnostic accuracy for obesity, and they are also components for metabolic syndrome [24]. Discrepancies in results suggest that prognostic ability of each index of obesity may differ by age, gender, and ethnic group. It is therefore believed that the discrete criteria to select a particular obesity index should be age, gender, and ethnic group specific. In this study, we observed that using WHR followed by WHtR yielded the highest prevalence of obesity compared to using BMI. It is with no doubt that the use of BMI alone may underutilize other equally obesity indicators such as WC, WHR, and WHtR.

Prevalence of obesity irrespective of the anthropometric measure used was higher among female than male participants. Being male or a female was significantly associated with obesity. Some authors [25–28] but not Gopalakrishnan et al. [29] have consistently reported that increased prevalence of obesity is associated with females than male participants. Findings of this study concur with the earlier authors. The proportion of obesity in females was extremely high in this study compared to current study by Mogre et al. [28]. The higher rate of obesity and overweight among the females is expected because there is a social perception which encourages fatness in the females in Ghanaian population. Females prefer being fat which is considered as a sign of good living in a society than having a slender body. Contraceptives usage is also known to cause the body to produce increased amount of fat in the females due to its appetite inducing nature, Reid et al. [30]. All these factors may account for the high prevalence of obesity among the women compared to the men.

The prevalence of obesity was also assessed in relation to the lifestyle characteristics of study participants. Interestingly, participants who ate snacks in between meals and ate late at night and those who were physically inactive and preferred fast foods were obese irrespective of the anthropometric measures used. However, among participants who had history of high alcoholic intake, the prevalence of obesity was significantly high when WHtR was used compared to WC, WHR, and BMI. This clearly shows that these lifestyle behaviours could be independent risk factors of obesity and thus early health advice on the need for diabetic patient to adjust to lifestyle modification may help prevent future risk of cardiovascular disease and cerebrovascular accidents (stroke).

The major limitation of this study is the use of hospital-based cross-sectional study design and thus the findings of this study cannot conclusively represent the general diabetic patients in Ghana. However, some findings of this study concur well with other previous studies.

## 5. Conclusion

There is an increased prevalence of obesity among diabetic patients. Being a female was significantly associated with increased risk of obesity. Taking of snacks in between meals, eating meals late at night, physical inactivity, excessive fast food intake, and alcoholic beverage intake were common causes of obesity among diabetic patients. Physical exercise and dietary measures to control body weight are lacking despite the desire to have appropriate body weight. There is a need and we strongly recommend patient education programs for the control of obesity among our patients. Again, public health awareness especially hospital-based awareness on healthy lifestyle and nutrition is essential in early management of diabetes. Using other anthropometric measurements such as WC, WHR, and WHtR in conjunction with BMI would be useful for better diagnosis of obesity condition.

## Figures and Tables

**Table 1:** Sociodemographic characteristics of type 2 diabetic patients.

Variable	Frequency	Percentage
Age (years) (mean ± SD)	51.14 ± 14.45	
<19	7	1.4%
20–39	91	16.8%
40–59	232	42.7%
60–79	198	36.4%
80–99	15	2.8%
Gender		
Male	232	42.7%
Female	311	57.3%
Occupation		
None	167	30.8%
Self-employed	304	55.9%
Govt employed	72	13.3%
Marital status		
Single	49	9.1%
Married	399	73.4%
Divorced	49	9.1%
Widowed	46	8.4%
Socioeconomic income (GHS)		
<500 (low)	292	53.8%
500–1000 (moderate)	205	37.8%
>1000 (high)	46	8.4%
Highest level of education		
None	61	11.2%
Primary	208	38.4%
Secondary	194	35.7%
Tertiary	80	14.7%
Ethnicity		
Akan	478	88.1%
Ga-Adangbe	4	0.7%
Ewe	15	2.8%
Mole-Dagbani	46	8.4%

**(a):** 

General knowledge	Adequate	Inadequate	Do not know
Understanding the meaning of obesity	391 (72.0%)	141 (25.9%)	11 (2.1%)
Ideal body weight	23 (4.2%)	307 (56.6%)	213 (39.2%)
Method of weight measurement	535 (98.6%)	8 (1.4%)	—

**(b):** 

Knowledge on causes and complications	*N*	Frequency
Causes of obesity		
Poor diet	418	76.9%
Physical inactivity	364	67.1%
Insufficient sleep/stress	4	0.7%
Family history of obesity	307	56.6%
Complications on obesity		
Hypertension	444	81.8%
Stroke	186	34.3%
Cancer	8	1.4%
Knowledge about management		
Dietary modification	471	86.7%
Physical activity	372	68.6%
Regular health check-up/medication	156	28.7%
Other lifestyle (alcohol intake, smoking, and sedentary activity)	—	—

Variables presented as frequency (percentages).

**Table 3:** Lifestyle characteristic of type 2 diabetic patients.

Lifestyle characteristics features	Frequency	Response
Diet		
Number of times meals are taken per day		
Twice	107	19.6%
Thrice	406	74.8%
Four	30	5.6%
Snack in between meals		
Yes	91	16.8%
No	452	83.2%
Taking meals late at night		
Yes	72	13.3%
No	471	86.7%
Time for taking late meal		
5 pm	69	12.6%
6 pm	338	62.2%
7 pm	125	23.1%
8 pm	11	2.1%
Physical activity		
Regular exercise		
Yes	133	24.5%
No	410	75.5%
Type of exercise		
Walking	310	57.1%
Jogging	233	42.9%
Gym	0	—
Number of weekly exercises		
Once	16	2.9%
Twice	186	34.3%
Thrice	31	5.7%
Daily	264	48.6%
Smoking lifestyle		
Yes	4	0.7%
No	539	99.3%
Alcohol intake		
Yes	15	2.8%
No	528	97.2%
Food preferences		
Soft drinks	288	53.1%
Fast food (burger, deep fried foods, and pizza)	213	39.2%
Red meat	118	21.7%
Butter, cheese, and cream	307	56.6%
Egg yolk	65	11.9%
Fiber rich foods	167	30.7%

**Table 4:** Anthropometric, clinical, and FBS levels characteristic of type 2 diabetic patients.

Parameters	Total	Male	Female	*p* value
Anthropometric index				
Weight (Kg)	64.37 ± 1.18	64.56 ± 1.77	64.24 ± 1.59	0.8959
Height (m)	1.65 ± 0.01	1.69 ± 0.01	1.61 ± 0.01	**<0.0001**
BMI (Kg/m^2^)	23.72 ± 0.42	22.31 ± 0.53	24.76 ± 0.60	**0.0038**
WC (cm)	74.85 ± 1.91	73.49 ± 2.82	75.84 ± 2.59	0.5452
HC (cm)	80.35 ± 2.05	77.90 ± 2.96	82.15 ± 2.81	0.3073
WHR	0.93 ± 0.01	0.93 ± 0.01	0.95 ± 0.01	0.1204
WHtR	0.46 ± 0.01	0.43 ± 0.02	0.47 ± 0.02	0.1001
Clinical characteristics				
SBP (mmHg)	130.8 ± 1.78	130.7 ± 2.39	131.0 ± 2.54	0.9319
DBP (mmHg)	79.30 ± 1.06	79.50 ± 1.45	79.15 ± 1.49	0.8693
FBS (mmol/l)	11.78 ± 0.53	13.52 ± 0.93	10.50 ± 0.58	**0.0044**

Mean ± SD. SD: standard deviation; BMI: body mass index; WC: waist circumference; HC: hip circumference; WHR: waist to hip ratio; WHtR: waist to height ratio; SBP: systolic blood pressure; DBP: diastolic blood pressure; FBS: fasting blood sugar.

**Table 5:** Prevalence of obesity according the gender.

Anthropometrics	Total	Male	Females	*p* value (*χ* ^2^, df)
	(*n* = 543)	(*n* = 235)	(*n* = 308)	
BMI classification				0.003 (13.90, 3)
Underweight	77 (14.1%)	106 (45.0%)	169 (55.0%)	
Normal	260 (47.9%)	131 (55.9%)	136 (44.1%)	
Overweight	126 (23.2%)	71 (30.3%)	215 (69.7%)	
Obese	80 (14.8)	34 (14.3%)	264 (85.7%)	
WC				<0.0001 (27.56, 2)
Normal	325 (59.9%)	141 (60.0%)	123 (40.0%)	
Overweight	77 (14.1%)	47 (20.0%)	246 (80.0%)	
Obese	142 (26.1)	32 (13.5%)	266 (86.5%)	
WHR				<0.0001 (76.57, 2)
Normal	46 (8.5%)	118 (50.0%)	154 (50.0%)	
Overweight	165 (30.3%)	224 (95.3%)	15 (4.7%)	
Obese	333 (61.3%)	35 (14.9%)	262 (85.1%)	
WHtR				0.0386 (0.7508, 1)
Normal	84 (59.2%)	38 (45.2%)	169 (54.8%)	
Obese	58 (40.8%)	22 (37.9%)	191 (62.1%)	

Values are presented in frequency with percentages in parenthesis. *χ* ^2^: Chi-square value; df: degree of freedom. *p* < 0.05 showed statistically significant difference. BMI: body mass index; WC: waist circumference; WHR: waist to hip ratio; WHtR: waist to height ratio.

**Table 6:** Association between lifestyle characteristic and obesity using BMI as an indicator.

Lifestyle	BMI				*χ* ^2^, df (*p* value)
	Underweight	Normal	Overweight	Obese	
	*n* = 76	*n* = 258	*n* = 125	*n* = 84	
Snack in between meals					30.13, 3 (<0.0001)
Yes (*n* = 95)	8 (8.3%)	40 (41.7%)	16 (16.7%)	31 (33.3%)	
No (*n* = 448)	69 (15.3%)	238 (53.1%)	92 (20.5%)	49 (11.0%)	
Taking meals late at night					23.39, 3 (<0.0001)
Yes (*n* = 76)	12 (15.8%)	32 (42.1%)	8 (10.5%)	24 (31.5%)	
No (*n* = 467)	64 (13.8%)	228 (48.8%)	118 (25.2%)	57 (12.2%)	
Regular exercise					43.60, 3 (<0.0001)
Yes (*n* = 133)	15 (11.4%)	95 (71.4%)	15 (11.4%)	7 (5.4%)	
No (*n* = 410)	61 (14.8%)	163 (39.8%)	110 (26.9%)	72 (17.6%)	
Fast food intake					3.074, 3 (0.3804)
Yes (*n* = 220)	30 (13.8%)	113 (51.7%)	46 (20.7%)	38 (17.2%)	
No (*n* = 323)	45 (14.1%)	144 (44.7%)	80 (24.7%)	42 (12.9%)	
Alcohol intake					51.08, 3 (<0.0001)
Yes (*n* = 15)	11 (75.0%)	0 (0)	0 (0)	4 (25.0%)	
No (*n* = 523)	64 (12.2%)	256 (48.9%)	124 (23.7%)	75 (14.4%)	

Values are presented in frequency with percentages in parenthesis. BMI: body mass index.

**Table 7:** Association between lifestyle characteristic and obesity using WC, WHR, and WHtR as indicators.

Lifestyles features	WC			WHR			WHtR	
	Normal	Overweight	Obese	Normal	Overweight	Obese	Normal	Obese
	*n* = 325	*n* = 76	*n* = 142	*n* = 46	*n* = 165	*n* = 330	*n* = 323	*n* = 220
Snack in between meals	0.6843, 2 (*p* = 0.7102)			12.84, 2 (*p* = 0.0016)			4.803, 1 (*p* = 0.0284)	
Yes (*n* = 95)	55 (58.3%)	16 (16.7%)	24 (25.0%)	16 (16.7%)	20 (20.8%)	59 (62.5%)	43 (45.8%)	13 (54.2%)
No (*n* = 448)	270 (60.2%)	61 (13.6%)	118 (26.3%)	30 (6.7%)	145 (32.2%)	273 (61.0%)	277 (61.9%)	171 (38.1%)
Taking meals late at night	1.929, 2 (*p* = 0.3812)			8.719, 2 (*p* = 0.0128)			4.882, 1 (*p* = 0.0271)	
Yes (*n* = 76)	40 (52.6%)	12 (15.8%)	24 (31.6%)	8 (10.5%)	12 (15.8%)	56 (73.7%)	36 (47.3%)	40 (52.6%)
No (*n* = 467)	284 (60.9%)	64 (13.8%)	118 (25.2%)	38 (8.1%)	152 (32.5%)	277 (59.3%)	284 (60.9%)	183 (39.1%)
Regular exercise	10.26, 2 (*p* = 0.0059)			1.486, 2 (*p* = 0.4757)			0.08455, 1 (*p* = 0.7712)	
Yes (*n* = 133)	95 (71.4%)	11 (8.6%)	27 (20.0%)	11 (8.6%)	46 (34.3%)	76 (57.1%)	80 (60.0%)	53 (40.0%)
No (*n* = 410)	228 (55.6%)	64 (15.7%)	114 (27.8%)	34 (8.3%)	118 (28.7%)	254 (62.0%)	239 (58.3%)	168 (40.7%)
Fast food intake	3.435, 2 (*p* = 0.1795)			17.27, 2 (*p* = 0.0002)			25.01, 1 (*p* < 0.0001)	
Yes (*n* = 220)	121 (55.2%)	34 (15.5%)	64 (29.3%)	31 (14.3%)	55 (25.0%)	141 (64.3%)	106 (48.2%)	122 (55.4%)
No (*n* = 323)	201 (62.3%)	42 (12.9%)	76 (23.5%)	15 (4.7%)	110 (34.1%)	194 (60.0%)	217 (67.1%)	103 (31.8%)
Alcohol intake	2.629, 2 (*p* = 0.2686)			8.748, 2 (*p* = 0.0126)			2.037, 1 (*p* = 0.1535)	
Yes (*n* = 15)	11 (75.0%)	—	4 (25.0%)	4 (25.0%)	7 (50.0%)	4 (25.0%)	1 (25.0%)	3 (75.0%)
No (*n* = 528)	314 (58.9%)	76 (14.4%)	138 (25.9%)	417 (7.9%)	156 (29.5%)	327 (61.9%)	315 (59.7%)	209 (39.6%)

Values are presented in frequency with percentages in parenthesis. Chi-square value (*χ* ^2^), df (*p* value). BMI: body mass index; WC: waist circumference; WHR: waist to hip ratio; WHtR: waist to height ratio.

**Table 8:** Association between level of knowledge of obesity and prevalence of obesity among type 2 diabetic patients.

Anthropometric indicators	General knowledge of obesity			*χ* ^2^, df (*p* value)
	Adequate	Inadequate	Do not know	
	(*n* = 391)	(*n* = 141)	(*n* = 11)	
BMI classification				9.917, 6 (0.1282)
Underweight	9 (2.3)	5 (3.5)	0 (0.0)	
Normal	101 (25.8)	38 (27.0)	3 (27.3)	
Overweight	198 (50.6)	73 (51.8)	2 (18.2)	
Obese	83 (21.2)	25 (17.7)	6 (54.5)	
WC				42.77, 4 (<0.0001)
Normal	113 (28.9)	57 (40.4)	2 (18.2)	
Overweight	187 (47.8)	33 (23.4)	0 (0.0)	
Obese	91 (23.3)	51 (36.2)	9 (81.8)	
WHR				13.26, 4 (0.0101)
Normal	121 (30.9)	51 (36.2)	3 (27.3)	
Overweight	193 (49.4)	49 (34.8)	3 (27.3)	
Obese	77 (19.7)	41 (29.1)	5 (45.5)	
WHtR				26.37, 2 (<0.0001)
Normal	303 (77.5)	85 (60.3)	3 (27.3)	
Obese	88 (22.5)	56 (39.3)	8 (72.7)	

Values are presented in frequency with percentages in parenthesis. Chi-square value (*χ* ^2^), df (*p* value). BMI: body mass index; WC: waist circumference; WHR: waist to hip ratio; WHtR: waist to height ratio.

## References

[1] Mumu S. J., Saleh F., Ara F., Haque M. R., Ali L. (2014). Awareness regarding risk factors of type 2 diabetes among individuals attending a tertiary-care hospital in Bangladesh: a cross-sectional study. *BMC Research Notes*.

[2] Hussain A., Claussen B., Ramachandran A., Williams R. (2007). Prevention of type 2 diabetes: a review. *Diabetes Research and Clinical Practice*.

[3] Buttar H. S., Li T., Ravi N. (2005). Prevention of cardiovascular diseases: role of exercise, dietary interventions, obesity and smoking cessation. *Experimental & Clinical Cardiology*.

[4] Merlotti C., Morabito A., Pontiroli A. E. (2014). Prevention of type 2 diabetes; a systematic review and meta-analysis of different intervention strategies. *Diabetes, Obesity and Metabolism*.

[5] Anderson J. W. (2003). Whole grains protect against atherosclerotic cardiovascular disease. *Proceedings of the Nutrition Society*.

[6] Buse J. B., Ginsberg H. N., Bakris G. L. (2007). Primary prevention of cardiovascular diseases in people with diabetes mellitus: a scientific statement from the American Heart Association and the American Diabetes Association. *Diabetes Care*.

[7] Ashrafi K. (2007). Obesity and the regulation of fat metabolism. *WormBook*.

[8] WHO (2000). *Obesity: Preventing and Managing the Global Epidemic*.

[9] Mugharbel K. M., Al-Mansouri M. A. (2003). Prevalence of obesity among type 2 diabetic patients in Al-Khobar primary health care centers. *Journal of Family & Community Medicine*.

[10] Grundy S. M., Benjamin I. J., Burke G. L. (1999). Diabetes and cardiovascular disease: a statement for healthcare professionals from the American Heart Association. *Circulation*.

[11] Habib S. S. (2012). Cardiovascular disease in diabetes: an enigma of dyslipidemia, thrombosis and inflammation. *Basic Research Journals of Medicine and Clinical Science*.

[12] Visscher T. L. S., Seidell J. C. (2001). The public health impact of obesity. *Annual Review of Public Health*.

[13] Alberti K. G. M. M., Zimmet P., Shaw J. (2007). International Diabetes Federation: a consensus on Type 2 diabetes prevention. *Diabetic Medicine*.

[14] WHO (1995). Physical status: the use of and interpretation of anthropometry. *Report of a WHO Expert Committee*.

[15] de Onis M., Habicht J.-P. (1996). Anthropometric reference data for international use: recommendations from a World Health Organization Expert Committee. *The American Journal of Clinical Nutrition*.

[16] Qidwai W., Azam S. I. (2004). Knowledge, attitude and practice regarding obesity among patients, at Aga Khan University Hospital, Karachi. *Journal of Ayub Medical College, Abbottabad*.

[17] Saleh F., Mumu S. J., Ara F., Ali L., Hossain S., Ahmed K. R. (2012). Knowledge, attitude and practice of type 2 diabetic patients regarding obesity: study in a tertiary care hospital in Bangladesh. *Journal of Public Health in Africa*.

[18] Saris W. H. M. (2003). Sugars, energy metabolism, and body weight control. *The American Journal of Clinical Nutrition*.

[19] Badruddin N., Basit A., Hydrie M. Z. I., Hakeem R. (2002). Knowledge, attitude and practices of patients visiting a diabetes care unit. *Pakistan Journal of Nutrition*.

[20] Webber J. (2003). Energy balance in obesity. *Proceedings of the Nutrition Society*.

[21] Akpinar E., Bashan I., Bozdemir N., Saatci E. (2007). Which is the best anthropometric technique to identify obesity: body mass index, waist circumference or waist-hip ratio?. *Collegium Antropologicum*.

[22] Bhurosy T., Jeewon R. (2014). Overweight and obesity epidemic in developing countries: a problem with diet, physical activity, or socioeconomic status?. *The Scientific World Journal*.

[23] Ashwell M., Gunn P., Gibson S. (2012). Waist-to-height ratio is a better screening tool than waist circumference and BMI for adult cardiometabolic risk factors: systematic review and meta-analysis. *Obesity Reviews*.

[24] Han T. S., Williams K., Sattar N., Hunt K. J., Lean M. E. J., Haffner S. M. (2002). Analysis of obesity and hyperinsulinemia in the development of metabolic syndrome: San Antonio Heart Study. *Obesity Research*.

[25] Onyechi U. A., Okolo A. C. (2008). Prevalence of obesity among undergraduate students, living in halls of residence, University of Nigeria, Nsukka Campus, Enugu State. *Animal Research International*.

[26] Oghagbon K. E., Odili V. U., Nwangwa E. K., Pender K. E. (2009). Body mass index and blood pressure pattern of students in a Nigerian University. *International Journal of Health Research*.

[27] van der A D. L., Nooyens A. C., van Duijnhoven F. J., Verschuren M. M., Boer J. M. (2014). All-cause mortality risk of metabolically healthy abdominal obese individuals: the EPIC-MORGEN study. *Obesity*.

[28] Mogre V., Nyaba R., Aleyira S. (2014). Lifestyle risk factors of general and abdominal obesity in students of the School of Medicine and Health Science of the University of Development Studies, Tamale, Ghana. *ISRN Obesity*.

[29] Gopalakrishnan S., Ganeshkumar P., Prakash M. V. S., Christopher A. V. (2012). Prevalence of overweight/obesity among the medical students, Malaysia. *The Medical Journal of Malaysia*.

[30] Reid I. R., Plank L. D., Evans M. C. (1992). Fat mass is an important determinant of whole body bone density in premenopausal women but not in men. *The Journal of Clinical Endocrinology & Metabolism*.

